# WEDGE-Net: Wavelet-Driven Memory-Efficient Anomaly Detection for Industrial Edge Computing

**DOI:** 10.3390/s26072154

**Published:** 2026-03-31

**Authors:** Joon-Min Park, Gye-Young Kim

**Affiliations:** School of Software, Soongsil University, Seoul 06978, Republic of Korea; aura1999@soongsil.ac.kr

**Keywords:** industrial edge computing, unsupervised anomaly detection, discrete wavelet transform, memory efficiency, noise robustness, real-time inspection

## Abstract

As deep learning-based Anomaly Detection (AD) transitions from theoretical research to industrial application, the focus is shifting towards operational efficiency and economic viability on edge devices. While recent studies have achieved remarkable detection accuracy on standard benchmarks, they often rely on heavy memory banks or complex backbones, which pose challenges for deployment in resource-constrained manufacturing environments. Furthermore, real-world inspection lines often present distinct challenges—such as environmental noise and strict latency requirements—that are not fully addressed by accuracy-centric metrics. To bridge the gap between high-performance research models and practical edge deployment, we introduce WEDGE-Net. Our approach is designed to balance structural precision with extreme memory efficiency. We decouple anomaly detection into two specialized streams: (1) a Frequency Stream (DWT) that physically filters out environmental noise to isolate structural defects, and (2) a Context Stream where a Semantic Module explicitly guides feature extraction to enforce object consistency. By synthesizing these two modalities, WEDGE-Net effectively suppresses high-frequency noise while enhancing structural-feature compactness. To validate operational stability, we conducted a robustness analysis of the ‘Tile’ category, which poses a challenging task for distinguishing defects from high-frequency textures. In this stress test, WEDGE-Net demonstrated superior resistance to environmental noise compared to conventional methods. Experimental results on the MVTec AD dataset demonstrate that WEDGE-Net achieves a mean image-level AUROC of 97.82% and an inference speed of 686.5 FPS (measured on an RTX 4090 GPU) under an extreme 1% memory-compression setting. Notably, our method demonstrates superior efficiency, achieving a 2.1× inference speedup over the widely adopted comparative model (PatchCore-10%) while maintaining competitive detection accuracy (e.g., 100% AUROC on Transistor). We hope this work serves as a practical reference for implementing real-time industrial inspection on resource-constrained edge devices.

## 1. Introduction

Automatic Optical Inspection (AOI) utilizing deep learning has become indispensable in modern manufacturing [1]. In real-world mass production scenarios, collecting normal samples is straightforward and abundant. However, obtaining sufficient abnormal samples remains a significant challenge due to the rarity of defects [2]. Consequently, Unsupervised Anomaly Detection (UAD), which trains exclusively on normal data to identify deviations, has become the standard approach in the industry. As comprehensively reviewed in recent surveys [3], deep learning-based UAD is rapidly evolving to address diverse industrial challenges, ranging from surface defect localization to logical anomaly detection.

State-of-the-art UAD methods, such as PatchCore [4], typically employ a memory bank mechanism that stores feature embeddings extracted from normal training images. While these methods achieve high detection accuracy, they often face scalability challenges related to memory overhead. Since the size of the memory bank grows linearly with the amount of training data, applying these models to large-scale industrial datasets results in substantial computational costs and high inference latency. This burden makes it difficult to deploy high-performance models on resource-constrained edge devices (e.g., Industrial PCs or embedded systems) commonly used on factory floors. To address such resource allocation and inference latency challenges in edge computing environments, various optimization techniques, including task-driven priority-aware computation offloading using deep reinforcement learning, have been actively explored [5].

To address this bottleneck, we introduce WEDGE-Net (Wavelet-Enhanced Dual-stream Guided Embedding Network), a frequency-aware architecture designed for extreme memory efficiency and environmental robustness [6]. We observe that most mass production lines (e.g., conveyor belts) maintain consistent object alignment, rendering rotation-invariant features redundant. Leveraging this controlled environment, WEDGE-Net utilizes a Discrete Wavelet Transform (DWT) stream to filter out high-frequency interferences—such as camera sensor noise or unstable lighting conditions—while the Semantic Module enhances structural distinctiveness. This synergistic design produces highly compact feature embeddings, allowing the normal manifold to be represented by a significantly sparse set of vectors.

**Figure 1:**
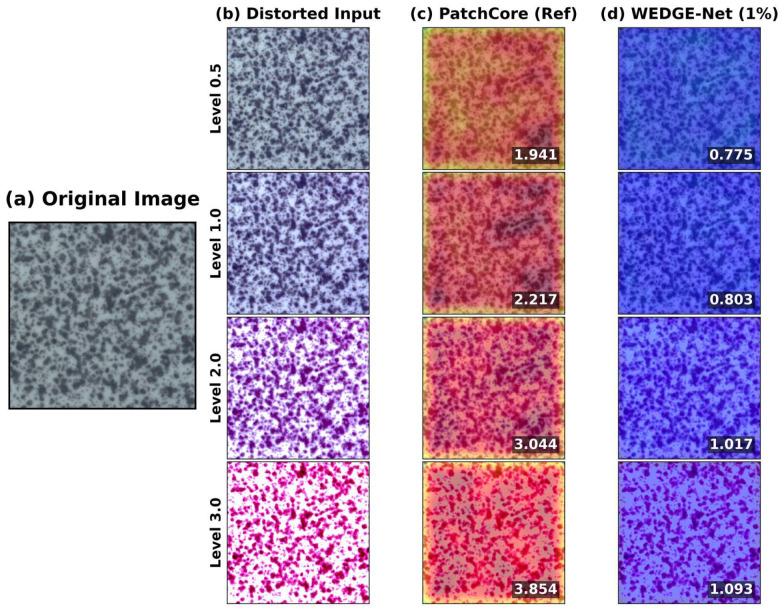
Qualitative robustness against domain shifts (*Tile*). (**a**) Original normal sample. (**b**) Inputs with increasing color jitter intensity. (**c**) PatchCore (10% memory) interprets global lighting changes as anomalies, yielding elevated scores. (**d**) WEDGE-Net (1% memory) demonstrates robust domain generalization by ignoring non-structural variations.

As qualitatively illustrated in Figure 1, we observe that under constrained memory settings (e.g., PatchCore-10%), the model may be influenced by global environmental shifts such as lighting changes. Specifically, as shown in Figure 1c, non-structural variations can lead to increased anomaly scores, visualized as red regions. In comparison, WEDGE-Net (1% memory, Figure 1d) is designed to mitigate such effects using frequency-based filtering, helping to maintain stability under these specific conditions. Consequently, our method offers a memory-efficient alternative that prioritizes operational stability in target categories.

## 2. Related Work

### 2.1. Reconstruction-Based Anomaly Detection

Early unsupervised anomaly detection methods primarily relied on reconstruction-based approaches, such as AutoEncoders (AEs) and Generative Adversarial Networks (GANs). The fundamental assumption is that a model trained only on normal samples will fail to accurately reconstruct anomalous regions. Methods like AE-SSIM [7], AnoGAN [8], and GANomaly [9] utilized pixel-wise reconstruction errors to localize defects. However, these methods often suffer from the generalization problem, in which the model creates a blurry reconstruction that inadvertently smoothens out high-frequency anomalies, leading to false negatives. To address this, recent works such as DRAEM [10] significantly improved performance by simulating synthetic anomalies and training a reconstruction network to restore the original image. Furthermore, diffusion-based models like AnoDDPM [11] have demonstrated superior generation quality. However, their iterative denoising process results in high inference latency, making them unsuitable for real-time edge applications.

### 2.2. Embedding-Based Anomaly Detection

To overcome the limitations of reconstruction models, embedding-based methods have emerged as the state of the art. These approaches extract feature vectors using pre-trained networks (e.g., ImageNet-trained ResNet [12]) and model the distribution of normal features. SPADE [13] and PaDiM [14] utilized multivariate Gaussian distributions to estimate the normality of local patches. More recently, PatchCore [4] achieved high performance by introducing a memory bank mechanism that stores a coreset of locally aware patch features.

To handle complex distributions, Normalizing Flow approaches like FastFlow [15] and CFLOW-AD [16] have been proposed to map features to a standard normal distribution. Alternatively, Reverse Distillation [17] employs a teacher-student framework to detect anomalies via discrepancy maps. While these methods excel at detecting textural anomalies, they often treat an image as a bag of independent patches. Consequently, detecting structural logical defects—such as a missing component or a rotated screw—remains a challenging task which often requires contextual awareness beyond local texture matching.

### 2.3. Feature Adaptation and Distillation-Based Anomaly Detection

Complementing high-capacity embedding models, recent studies have introduced lightweight architectures optimized for edge deployment through feature adaptation and knowledge distillation. SimpleNet [18] proposed a lightweight feature adapter to reduce the complexity of feature matching, while EfficientAD [19] introduced a student-teacher network optimized for extreme speed. While these methods achieve high throughput, they often rely on large-scale training data to establish robust decision boundaries. In contrast, WEDGE-Net explicitly addresses the data-efficiency problem by leveraging wavelet-domain priors, achieving competitive performance even with a compact memory bank, making it highly suitable for resource-constrained industrial environments.

### 2.4. Frequency-Domain Analysis in Deep Learning

Frequency domain analysis, particularly using the Fourier Transform or the Wavelet Transform, has been widely used in signal processing but is relatively underexplored in deep learning-based anomaly detection. Some recent works have attempted to bridge this gap. WaveCNet [20] integrated wavelet pooling into CNNs to reduce spatial redundancy while preserving structural information, and DW-GAN [21] utilized dual-wavelet discriminators to enhance high-frequency detail capture.

However, most existing methods utilize frequency transforms primarily as a pre-processing step [22] or a loss function constraint [23]. We identified this as a critical research gap. Recognizing that environmental noise and structural defects typically occupy distinct frequency bands, we hypothesized that explicitly disentangling these components could be the breakthrough for robust industrial inspection. To the best of our knowledge, WEDGE-Net is among the first architectures to explicitly integrate a wavelet-driven dual-stream mechanism for memory-efficient anomaly detection in edge computing.

While recent anomaly detection studies have explored frequency domain analysis, they often involve computationally intensive operations. Our approach differs in that it avoids complex frequency operations; instead, we focus on a lightweight dual-stream integration specifically optimized for memory-constrained industrial edge deployment.

## 3. Materials and Methods

### 3.1. Overall Architecture

**Figure 2:**
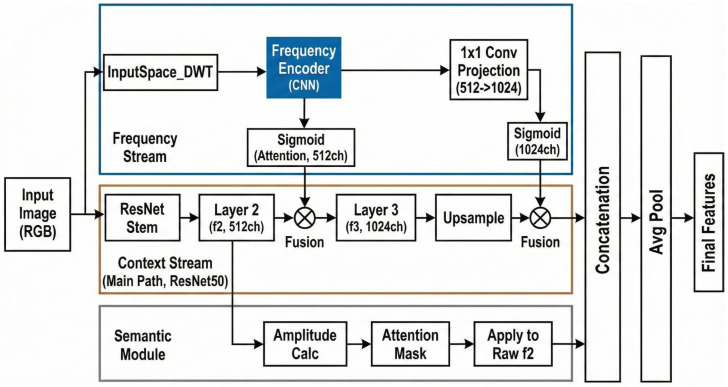
Overview of WEDGE-Net. The architecture consists of two parallel streams: (1) The Context Stream (RGB) extracts semantic features using a ResNet backbone with a Semantic Module. (2) The Frequency Stream (DWT) decomposes the image and directly processes the unified sub-bands through the Frequency Encoder to generate an attention map that modulates the semantic features, suppressing noise and enhancing structural defects.

The proposed WEDGE-Net architecture is illustrated in Figure 2. The overall workflow proceeds in three logical steps: feature extraction, frequency-aware fusion, and anomaly scoring.

First, an input image $I\in R^{H\times W\times C}$ is fed into two specialized streams. The Context Stream ($S_{RGB}$) utilizes a ResNet backbone to extract high-level representations. Integrated within this stream is the Semantic Module, which captures global object consistency to prevent semantic ambiguity. Simultaneously, the Frequency Stream ($S_{Freq}$) leverages the Discrete Wavelet Transform (DWT) to construct a noise-robust guidance map. By aggregating the total spectral energy from all frequency sub-bands, it emphasizes dominant structural components while suppressing incoherent environmental noise.

Second, the features from both streams represent different modalities of the same object. To synthesize them, the Frequency Encoder generates a spatial attention map, which acts as a gating mechanism. This map is element-wise multiplied with the context features in the Fusion Module, effectively suppressing background noise while highlighting structural boundaries.

Finally, the fused feature vectors are aggregated and stored in a memory bank. During inference, the anomaly score is calculated by measuring the distance between the query image’s features and the stored normal features. This dual-stream design ensures that the model makes decisions based on both semantic context and structural integrity.

In our architecture, the fusion of frequency (DWT) and context features naturally increases the dimensionality of the feature vectors (D=2048). To balance this computational load and ensure high-speed inference, we deliberately employed the standard ResNet-50 backbone instead of the heavier WideResNet-50 typically used in comparable methods [4]. While the wider backbone captures finer details, WEDGE-Net compensates for the lighter backbone by explicitly incorporating frequency-aware structural features. This design choice allows us to achieve high accuracy with a significantly lighter and faster underlying network.

### 3.2. Frequency-Aware Stream

To explicitly capture structural anomalies while suppressing noise, we introduce a dedicated Frequency Stream. This stream operates by decomposing the image into frequency sub-bands and generating a robust guidance map.

#### 3.2.1. Discrete Wavelet Transform (DWT)

Unlike standard CNNs that process raw pixels, our Frequency Stream leverages the 2D Discrete Wavelet Transform (DWT) to decompose the input image *I* into frequency sub-bands [24]. We tuilize the Haar wavelet kernel due to its superior suitability for industrial edge computing; unlike complex kernels that require floating-point multiplications, Haar relies on simple addition and subtraction, effectively minimizing hardware gate count and latency in FPGA or ASIC implementations.

The level-1 decomposition of input *I* results in four sub-bands by applying low-pass (*L*) and high-pass (*H*) filters along the rows and columns. Let (i,j) denote the pixel indices in the downsampled sub-bands. The Haar DWT operation is defined by the following linear combinations of the input image pixels:

(1) $$I_{LL}\left(i,j\right)=\frac{1}{2}\left(I_{2i,2j}+I_{2i+1,2j}+I_{2i,2j+1}+I_{2i+1,2j+1}\right)$$

(2) $$I_{LH}\left(i,j\right)=\frac{1}{2}\left(-I_{2i,2j}-I_{2i+1,2j}+I_{2i,2j+1}+I_{2i+1,2j+1}\right)$$

(3) $$I_{HL}\left(i,j\right)=\frac{1}{2}\left(-I_{2i,2j}+I_{2i+1,2j}-I_{2i,2j+1}+I_{2i+1,2j+1}\right)$$

(4) $$I_{HH}\left(i,j\right)=\frac{1}{2}\left(I_{2i,2j}-I_{2i+1,2j}-I_{2i,2j+1}+I_{2i+1,2j+1}\right)$$

As formulated above, $I_{LL}$ represents the approximation (low-pass) component that captures the global structural topology. In contrast, $I_{LH},I_{HL},$ and $I_{HH}$ correspond to the horizontal, vertical, and diagonal detail (high-pass) components, respectively. By explicitly disentangling these frequency bands, WEDGE-Net can effectively isolate structural edges while suppressing stochastic environmental noise that typically resides in the high-frequency sub-bands.

#### 3.2.2. Guidance Map Construction

To construct a comprehensive guidance map *G* that captures both the global topology and local structural details, we aggregate the information from all decomposed sub-bands. Rather than treating the low-frequency approximation ($I_{LL}$) and high-frequency details ($I_{LH},I_{HL},I_{HH}$) separately, we compute the total spectral energy at each spatial location:

(5) $$G=\sqrt{{\left(I_{LL}\right)}^{2}+{\left(I_{LH}\right)}^{2}+{\left(I_{HL}\right)}^{2}+{\left(I_{HH}\right)}^{2}}$$

This unified formulation ensures that the guidance map retains the object’s overall shape (dominated by the high-energy $I_{LL}$ component) while simultaneously incorporating sharp boundary discontinuities preserved in the high-frequency bands. Specifically, during our initial design phase, we empirically evaluated various learnable weighting schemes across the sub-bands. Because these alternatives yielded no significant performance gain over the unweighted spectral energy computation, we adopted this parameter-free formulation to strictly minimize computational overhead for edge deployment.

#### 3.2.3. Frequency Encoder and Projection

The guidance map *G* is processed by a Frequency Encoder to generate a frequency-aware attention map. First, local frequency features $F_{freq}$ are extracted via a lightweight CNN. Specifically, the 1-channel guidance map *G* passes through an initial 3×3 convolutional layer that expands the depth to 64 channels, followed by a ReLU activation. A subsequent 3×3 convolution further expands the features to 512 channels, followed by another ReLU activation. To maintain spatial resolution, all convolutional layers use a stride of 1 with appropriate zero-padding, and no pooling or downsampling operations are applied:

(6) $$F_{freq}=\mathrm{ReLU}\left({\mathrm{Conv}}_{3\times 3}\left(\mathrm{ReLU}\left({\mathrm{Conv}}_{3\times 3}\left(G\right)\right)\right)\right)$$

Subsequently, to fuse this information with the backbone features (ResNet Layer 3, exactly 1024 channels), we apply a 1×1 pointwise convolution. This projection layer acts as a dimension matching mechanism, mapping the 512-channel frequency features to the target 1024-channel space:

(7) $$F_{proj}=W_{1\times 1}*F_{freq}+b$$

Finally, the attention map $A_{freq}$ is obtained via the Sigmoid activation:

(8) $$A_{freq}=\sigma \left(F_{proj}\right)=\frac{1}{1+e^{-F_{proj}}}$$

### 3.3. Dual-Stream Fusion Mechanism

The core contribution of WEDGE-Net is the fusion of frequency attention into the backbone. Let $F_{l}$ be the feature map of the ResNet backbone at layer *l*. The modulated feature map $F_{l}^{\prime }$ is computed via element-wise multiplication:

(9) $$F_{l}^{\prime }=F_{l}⊗\left(1+A_{freq}\right)$$

Here, ⊗ denotes element-wise multiplication. We employ a residual attention mechanism, represented by the term $\left(1+A_{freq}\right)$, instead of standard softmax normalization. Unlike softmax, which produces a competitive distribution over features, the residual formulation preserves the original context while enabling adaptive feature modulation. Specifically, the constant term maintains the backbone features $\left(F_{l}\right)$, while $A_{freq}$ enhances structurally informative regions.

### 3.4. Feature Aggregation and Memory Bank

The final stage of WEDGE-Net involves aggregating the modulated features and constructing a coreset memory bank for anomaly scoring.

#### 3.4.1. Backbone Selection: Standard vs. Wide

A critical design choice in WEDGE-Net is the selection of the backbone network. Existing state-of-the-art methods typically rely on Wide ResNet-50-2 (approx. 68 million parameters) to ensure sufficient feature density. While effective, the substantial computational cost of Wide ResNet presents a challenge for real-time deployment on edge devices.

In contrast, WEDGE-Net utilizes the lightweight Standard ResNet-50 (approx. 25 million parameters). We hypothesize that the reduced capacity of the standard backbone can be effectively compensated by our dual-stream mechanism: the Frequency Stream filters out noise, and the Context Stream enhances structural distinctiveness. This strategic offloading allows us to achieve high-performance anomaly detection with a significantly lighter architecture (2.7× fewer parameters).

#### 3.4.2. Semantic Module for Global Attention

To further refine the feature map extracted from the backbone (specifically, ResNet Layer 2), we introduce a Semantic Module. Unlike local patch-based features, which treat the background and object equally, this module computes a global attention mask to highlight the object’s silhouette. The masking process consists of the following sequential steps.

First, the channel-wise mean amplitude of the absolute feature values is calculated to form an initial spatial map $M_{amp}$:

(10) $$M_{amp}=\frac{1}{C}\sum_{k=1}^{C}\left|F_{l}^{k}\right|$$

Second, to bound the attention weights and ensure stable modulation, a min-max normalization is applied to generate the final semantic mask $M_{sem}$ with values ranging from 0 to 1:

(11) $$M_{sem}=\frac{M_{amp}-\min\left(M_{amp}\right)}{\max\left(M_{amp}\right)-\min\left(M_{amp}\right)+\epsilon }$$

where ϵ is a small constant ($10^{-6}$) to prevent division by zero.

Third, the raw backbone features $F_{l}$ are L2-normalized along the channel dimension. The semantic mask $M_{sem}$ is then applied via element-wise multiplication to weight the normalized features spatially.

Finally, this semantically masked feature representation is concatenated with the frequency-fused context features. This mechanism acts as a spatial filter, suppressing irrelevant background textures and guiding the model’s focus toward the dominant structural regions of the target object.

#### 3.4.3. Memory Bank with Greedy Coreset Subsampling

To ensure efficient inference, we compress the memory bank M using greedy coreset subsampling [4]. This method approximates the original feature distribution by iteratively selecting a subset $M_{core}$ that minimizes the maximum distance to the remaining features:

(12) $$M_{core}=\underset{S\subset M,|S|=N}{\arg\min}\;\max_{x\in M}\min_{s\in S}{∥x-s∥}_{2}$$

where *N* denotes the target memory size. In this study, we adopt a strict sampling ratio of 1% (N=0.01|M|), prioritizing inference efficiency for edge deployment while maintaining a representative feature distribution (validation of this ratio is provided in Section 4.6.3).

#### 3.4.4. Memory Bank Construction Procedure

The complete memory bank construction pipeline of WEDGE-Net is summarized in Algorithm 1. The process integrates the dual-stream feature extraction, frequency-aware fusion, and memory bank construction into a unified framework.

For reproducibility, the source code and implementation details are available at https://github.com/aura1999jmpark/WEDGE-Net (accessed on 5 February 2026). The repository provides the code used in our experiments and will be maintained with updates for documentation and usability.
**Algorithm 1** Memory Bank Construction of WEDGE-Net**Require:** Training images $D_{train}$, Coreset ratio ϵ (e.g., 0.1)
**Ensure:** Compressed Memory Bank $M_{core}$
   1: Initialize M←∅, Encoders $E_{ctx},E_{freq}$
   2: **for** each image $x\in D_{train}$ **do**

   3:        $x_{LL},\ldots ,x_{HH}\leftarrow \mathrm{DWT}\left(x\right)$                ▷ Frequency Stream

   4:        $M_{attn}\leftarrow \mathrm{Sigmoid}\left(E_{freq}\left(x_{LL},\ldots ,x_{HH}\right)\right)$
   5:        $F_{ctx}\leftarrow E_{ctx}\left(x\right)$                      ▷ Context Stream
   6:        $F_{fused}\leftarrow F_{ctx}⊗\left(1+\mathrm{Resize}\left(M_{attn}\right)\right)$          ▷ Freq-aware Fusion
   7:        $M\leftarrow M\cup \{F_{fused}\}$
   8: **end for**
   9: $M_{core}\leftarrow \mathrm{CoresetSampling}\left(M,\epsilon \right)$          ▷ Memory Compression
  10: **return** $M_{core}$


## 4. Experiments and Analysis

### 4.1. Experimental Setup

#### 4.1.1. Dataset and Baselines

We utilized the MVTec AD dataset [25], the standard benchmark for unsupervised anomaly detection, consisting of 5354 high-resolution images across 15 diverse industrial categories. The training set contains only normal images to simulate real-world scenarios, while the test set includes various structural and textural defects. For comparative analysis, we benchmarked WEDGE-Net against state-of-the-art methods, specifically selecting PatchCore [4] as the primary baseline due to its strong and widely reported performance. Our evaluation focuses on verifying that WEDGE-Net can achieve competitive accuracy using a standard ResNet-50 backbone, contrasting with PatchCore’s heavier WideResNet-50 architecture. Performance is primarily evaluated using the standard image-level AUROC metric, reflecting the practical objective of rapid binary decision-making in industrial inspection workflows. While pixel-level evaluation is valuable for detailed defect analysis, such analysis is typically performed in subsequent inspection stages rather than during the initial anomaly screening process. For completeness, we additionally report pixel-level AUROC and Per-Region Overlap (PRO) scores in the Supplementary Materials.

#### 4.1.2. Implementation Details

WEDGE-Net was implemented in PyTorch (version 2.0.1) using a ResNet-50 backbone pre-trained on ImageNet. To balance efficiency and semantic representation, we extracted features from intermediate layers (Layer 2 and Layer 3). Input images were resized to 256×256 and subsequently center-cropped to 224×224 without additional data augmentation. Feature extraction was performed using a batch size of 32. To ensure deterministic operations and full reproducibility during the memory bank construction, the random seed was strictly fixed at 42. DWT was applied for frequency decomposition.

Crucially, for the memory bank, we adopted a deliberately aggressive greedy coreset sampling ratio of 10% (compared to the typical 10–25% in prior arts) to demonstrate the compactness of our frequency-aware features. All experiments were conducted on a single NVIDIA GeForce RTX 4090 GPU, measuring inference speed (FPS) to assess edge deployment feasibility.

### 4.2. Operational Characteristics and Limitations

Before presenting quantitative benchmarks, we first analyze the qualitative operational characteristics of WEDGE-Net to clearly establish its functional boundaries and design rationale.

**Figure 3:**
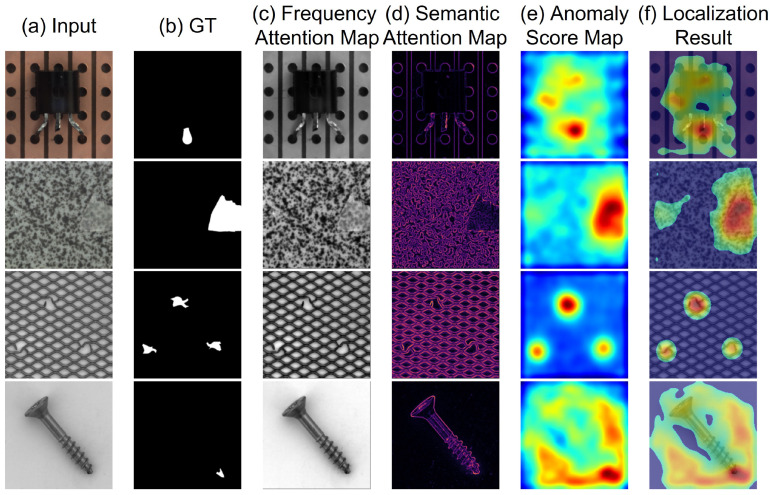
Visualization of intermediate representations for a structural object (*Transistor*) and a texture-centric one (*Grid*/*Screw*). (**a**) Test image. (**b**) Defect ground truth. (**c**) Frequency attention map isolating structural edges. (**d**) Semantic attention map capturing global shape. Red regions in (**c**,**d**) indicate higher anomaly scores. This highlights WEDGE-Net’s frequency-aware anomaly distinction.

To clearly illustrate the operational boundaries of WEDGE-Net, we present its capability profile in Figure 3.

- Strength (Structural Robustness): By leveraging DWT, the model explicitly focuses on dominant structural edges (e.g., boundaries of a Transistor). This makes it highly robust to environmental noise and effective for aligned structural objects, as confirmed by our noise robustness experiments.
- Limitation (Rotation & Texture): Conversely, this structural specialization introduces a trade-off. Since standard DWT kernels are not rotation-invariant, the model shows reduced sensitivity to unaligned objects (e.g., rotated Screws). Furthermore, in purely textural categories (e.g., Grid) where unique global structures are absent, the frequency stream provides limited gain compared to texture-specialized models. This design choice prioritizes precision in controlled manufacturing environments over general-purpose versatility.

### 4.3. Comparison with State-of-the-Art

We demonstrate the effectiveness of WEDGE-Net by comparing it with the current state-of-the-art method, PatchCore [4], on the full MVTec AD dataset [25]. Table 1 presents a comprehensive comparison across all 15 categories. In contrast to PatchCore, which prioritizes feature capacity using a WideResNet-50 backbone, our results are reported across four memory configurations (100%, 10%, 1%, and extreme 0.1%) using a lightweight ResNet-50 to highlight efficiency.

*Performance Analysis by Domain:* To explicitly analyze the operational characteristics of our frequency-aware architecture, we grouped the results into our Target Domain (Aligned Structure & Noise-Sensitive) and the remaining Texture/Structure categories.

- Target Domain (Robustness Verification): In categories requiring strict structural alignment (*Transistor, Bottle, Cable*) and noise robustness (*Tile*), WEDGE-Net demonstrates superior performance. Notably, even with an extreme compression ratio of 1%, our model achieves 100.00% AUROC on *Transistor* and *Bottle*, and surpasses the official PatchCore baseline on *Tile* (98.77% vs 98.70%) and *Cable* (99.66% vs 99.50%). Furthermore, our stress test at the 0.1% ratio reveals that *Transistor* and *Bottle* maintain near-perfect accuracy, confirming that our frequency stream effectively filters out environmental noise while preserving critical defect features.
- Generalization on Texture & Structure: In complex texture categories such as *Grid* and *Screw*, which lack global structural consistency, a performance gap exists compared to the full-capacity baseline. However, it is crucial to observe the *’Less is More’* phenomenon in our model. As shown in Table 1, as the memory size decreases from 100% to 1%, the performance in these challenging categories paradoxically improves (e.g., *Grid*: 89.72% → 91.14%, *Screw*: 86.74% → 89.20%). This indicates that the greedy coreset sampling acts as an effective de-noising filter, discarding redundant features that confuse the decision boundary.

*Overall Assessment:* WEDGE-Net (1%) achieves a global average AUROC of 97.82%, which is highly competitive with the full-memory PatchCore (99.1%). We acknowledge an architectural difference between WEDGE-Net (ResNet-50 at a 1% coreset) and the primary reference method, PatchCore (WideResNet-50). WEDGE-Net’s dual-stream architecture concatenates highly heterogeneous features, inherently producing a 2048-dimensional vector. Our initial attempts to apply feature projection for dimensionality reduction led to severe information loss and degraded performance, making the 2048-dimensional representation empirically necessary. Because this high-dimensional structure naturally increases latency under identical backbone conditions, we deliberately adopted a lighter backbone combined with an aggressive memory reduction strategy (a 1% coreset) to satisfy the strict requirements of edge environments. As shown in Table 1, this optimization successfully accelerates inference speeds (approx. 686 FPS) while maintaining a higher average detection performance than PatchCore evaluated at a 10% ratio, presenting a highly efficient operational trade-off. Furthermore, the fact that our 1% model outperforms our own 100% model (97.82% vs 97.52%) provides strong empirical evidence for the data efficiency of the proposed architecture.

### 4.4. Efficiency Benchmarking

To validate the feasibility of edge deployment, we evaluated the inference efficiency using an NVIDIA RTX 4090. We benchmarked against the official PatchCore [4] and its memory-optimized variant (10% memory).

Table 2 summarizes the results. WEDGE-Net (1% memory) operates at an inference speed of 686.5 FPS (1.46 ms). Compared to the optimized PatchCore setting (10% memory, 3.0 ms), this corresponds to a 2.1× reduction in latency. It is important to note that this speedup is not derived from an architectural acceleration of the feature extractor itself, but rather from the significant reduction in memory search cost. While PatchCore typically requires at least 10% memory to maintain reasonable accuracy, WEDGE-Net preserves its performance even at extreme compression rates of 1% or even 0.1% (as shown in Table 1).

To evaluate the practical deployment feasibility in GPU-less Industrial PC (IPC) environments, we conducted an additional CPU-only inference experiment using an Intel Core i9-14900K processor. Under this CPU-only setting, the comparative model exhibited a primary computational bottleneck due to its heavy WideResNet-50 backbone. Even when extreme memory compression (1%) was applied to the comparative model to minimize the k-NN search cost, its inference speed was limited to approximately 33.4 FPS due to the inherent feature-extraction latency. In contrast, WEDGE-Net utilizes a lighter ResNet-50 backbone. Combined with our primary 1% memory setting, WEDGE-Net achieves approximately 39.4 FPS, and further accelerates to 55.5 FPS at a 0.1% setting. These empirical results suggest that WEDGE-Net’s architectural design, pairing a lightweight backbone with aggressive memory compression, provides a definitive speed advantage for real-world edge deployment.

**Figure 4:**
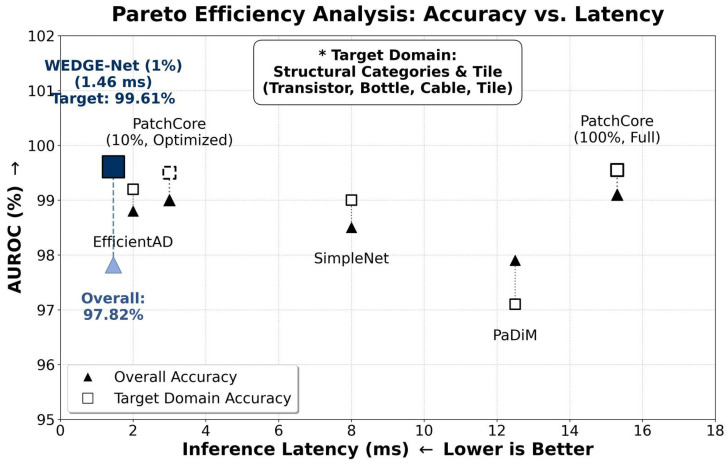
Performance Trade-off Analysis. Accuracy and latency metrics for various methods (including EfficientAD, PaDiM, and SimpleNet) are integrated to visualize their relative efficiency. Latency values for prior methods were projected relative to the measured PatchCore benchmark on an RTX 4090. WEDGE-Net (1% Memory) demonstrates a favorable trade-off, achieving the highest target domain accuracy (99.61%) with a minimal latency of 1.46 ms. Asterisks (*) indicate the target domain categories.

To provide a holistic view, Figure 4 visualizes the latency-accuracy trade-off across various state-of-the-art methods. Since reproducing every prior method on the same hardware is impractical, we established the measured latency of PatchCore on our RTX 4090 as the anchor point. We then projected the inference speeds of the other evaluated methods (e.g., PaDiM, SimpleNet, EfficientAD) based on the relative latency ratios reported in their respective original publications. As illustrated in the figure, WEDGE-Net is positioned in the top-left region, demonstrating that it effectively retains high accuracy under low-latency constraints required for real-time industrial inspection.

### 4.5. Environmental Robustness Analysis

In practical industrial settings, sensory noise is often unavoidable [26,27]. To verify the robustness of WEDGE-Net, we conducted comprehensive experiments analyzing both noise resistance and domain-shift stability. We specifically selected the ’Tile’ category as the primary benchmark. As a representative texture class rich in high-frequency patterns, ’Tile’ presents a challenging scenario for distinguishing between real defects and additive noise. Demonstrating robustness on ’Tile’ thus implies stability across easier object-centric categories.

#### 4.5.1. Noise Robustness

**Figure 5:**
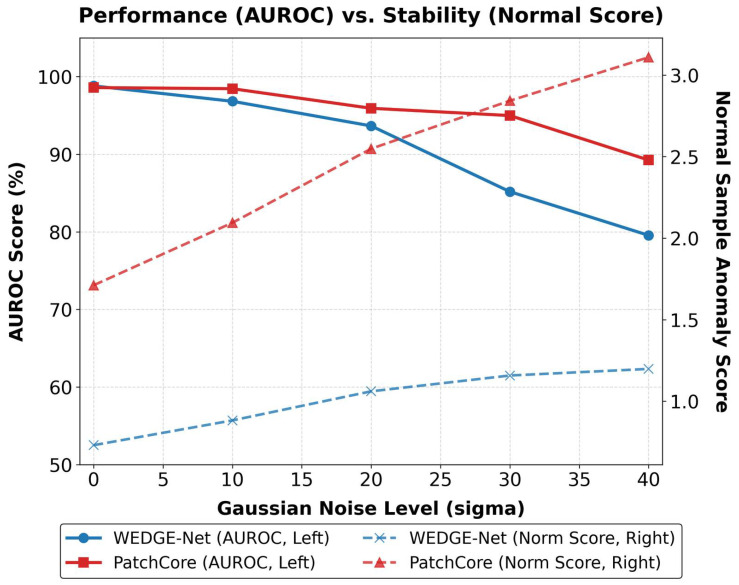
Quantitative Analysis of Noise Robustness (*Tile*). We visualize both detection performance (AUROC, solid lines, left axis) and stability (Normal Sample Score, dashed lines, right axis). While PatchCore shows high AUROC, its anomaly scores for normal samples increase sharply with noise (red dashed line), indicating potential instability. In contrast, WEDGE-Net maintains low and stable scores (blue dashed line), validating its resilience to environmental noise.

We compared WEDGE-Net (1% memory) against PatchCore (10% memory) under varying noise levels (σ∈{0,…,40}). Figure 5 presents a dual-axis analysis to evaluate both detection accuracy (AUROC, left axis) and operational stability (Normal Sample Score, right axis). While PatchCore maintains high AUROC scores, the right axis reveals a critical insight regarding industrial suitability:

- Sensitivity to High-Frequency Noise (PatchCore): As noise intensity increases, the baseline shows a correlation between high-frequency perturbations and anomaly scores. This is evidenced by the rise in anomaly scores for normal samples (red dashed line, right axis). In production, such score variations may necessitate careful threshold tuning to maintain optimal performance.
- Operational Stability (WEDGE-Net): Despite the compact memory setting, WEDGE-Net effectively filters noise, maintaining consistently low anomaly scores on normal samples (blue dashed line, right axis). Although the AUROC gap narrows in extreme noise, our method’s ability to decouple noise from structural defects ensures that normal products are not misclassified.

**Figure 6:**
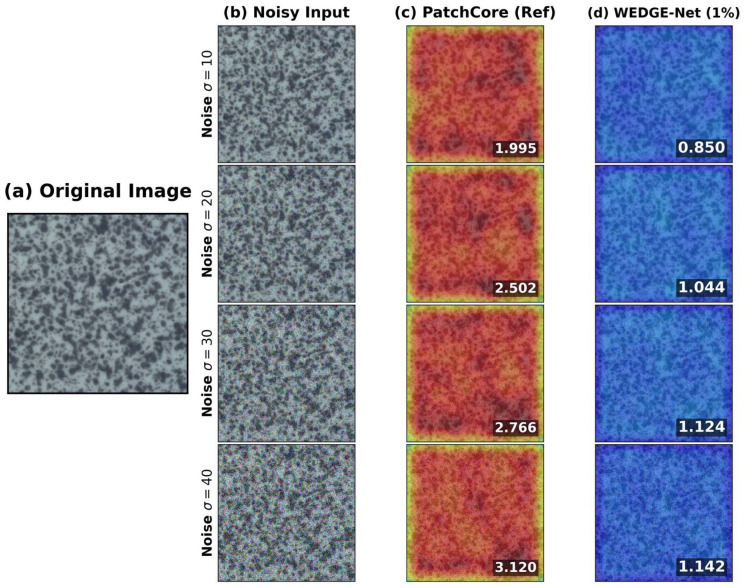
Qualitative Noise Robustness. (**a**) Original normal sample. (**b**) Inputs with increasing Gaussian noise (σ∈{10,…,40}). (**c**) PatchCore (10% memory) exhibits high sensitivity to noise. (**d**) WEDGE-Net (1% memory) effectively suppresses noise artifacts, maintaining stability.

This stability is visually confirmed in Figure 6. The baseline (Figure 6c) interprets noise as anomalies, exhibiting elevated scores (red regions). In contrast, WEDGE-Net (Figure 6d) effectively suppresses these artifacts, maintaining low scores (blue regions). This suggests that WEDGE-Net is well-suited for industrial inspection, mitigating noise-induced fluctuations and alleviating the need for frequent threshold recalibration.

#### 4.5.2. Domain Shift Robustness

Beyond pixel-level noise, industrial environments often suffer from complex imaging conditions such as global lighting changes or environmental variations [28]. To evaluate stability against such variations, we simulated severe color jittering (intensity factor ∈[0,4.0]), comparing WEDGE-Net (1% memory) against the PatchCore (10% memory) comparative model.

**Figure 7:**
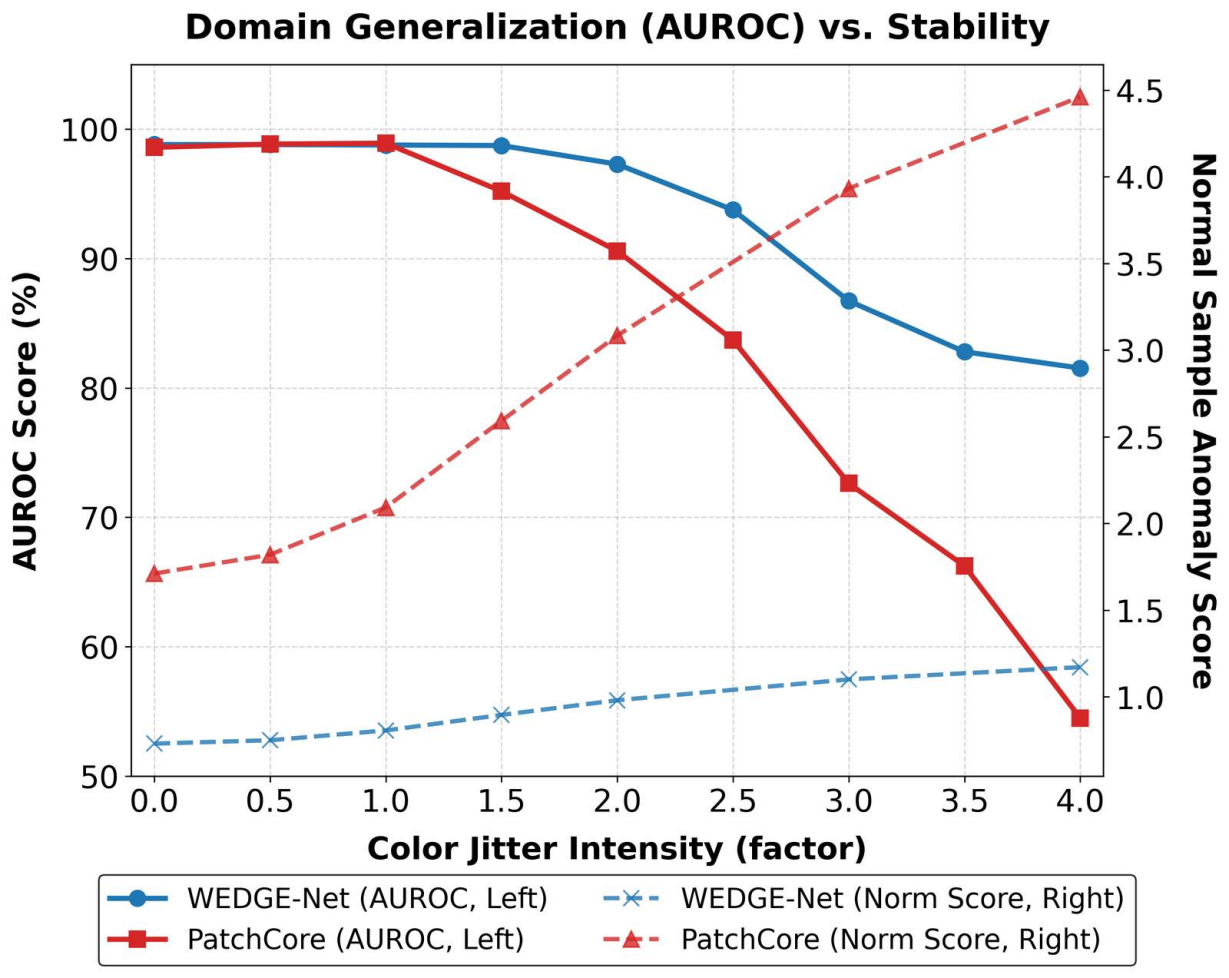
Quantitative Analysis of Domain Shift Robustness (Color Jitter). We compare performance under severe lighting variations (intensity up to 4.0). PatchCore (10% memory) exhibits sensitivity to domain shifts, leading to performance degradation and increased normal scores (red lines). WEDGE-Net (1% memory), however, retains stable performance and low normal scores (blue lines), demonstrating superior domain generalization.

As illustrated in Figure 7, both models exhibit high stability under minor shifts (intensity ≤1.0). However, a noticeable performance divergence emerges as environmental distortion intensifies. PatchCore (10% memory) due to its rigid memory-matching tends to interpret global lighting changes as anomalies. This results in a decrease in AUROC and a simultaneous rise in normal sample scores (red dashed line). In contrast, WEDGE-Net (1% memory) separates structural features from global low-frequency variations using its frequency-aware mechanism. Consequently, it maintains robust AUROC performance and keeps false-positive responses low (blue dashed line) even at intensity 4.0. This confirms that our approach effectively generalizes across domain shifts while preserving defect detection capabilities, as qualitatively demonstrated in Figure 1.

### 4.6. Ablation Study

To validate the architectural choices of WEDGE-Net, we focused on three critical perspectives: the contribution of the Semantic Module, the selection of the wavelet kernel, and the impact of the sampling strategy.

#### 4.6.1. Effectiveness of Dual-Stream Design

We investigated the validity of our architectural design by comparing the performance of the Frequency Stream alone (Semantic OFF) versus the complete WEDGE-Net (Semantic ON) at a 10% memory ratio.

Table 3 summarizes the results. While the Frequency Stream effectively suppresses noise (achieving 99.42% on Tile) due to the robust spectral decomposition, relying solely on it limits the model’s ability to capture global semantic understanding. The Semantic Module addresses this by recovering high-level feature representations.

Notably, in the *Screw* category, enabling the Semantic Module results in a substantial performance improvement from 71.45% to 88.03% (+16.58%). Furthermore, object-centric categories like *Capsule* also see a steady improvement (+1.23%). This confirms the complementary nature of our dual-stream design: the Semantic Module captures the object’s semantic identity, while the Frequency Stream concurrently filters out environmental noise, ensuring robust anomaly detection.

#### 4.6.2. Wavelet Kernel Selection

We empirically validated the choice of the wavelet kernel. The primary motivation for adopting the Haar wavelet is its high computational efficiency. We quantified the computational cost difference between the two kernels using the DWT MAC (Multiply-Accumulate operations) metric, as reported in Table 4. While the Haar wavelet requires approximately 12.85 million MACs, Biorthogonal 2.2 demands 80.28 million MACs (a 6.2× increase in arithmetic complexity). As presented in Table 4, despite this minimal computational footprint, the Haar wavelet maintains robust detection accuracy. It yields a marginal improvement in the global average (+0.87% at 1% memory) over the Biorthogonal 2.2 kernel by effectively preserving sharp structural discontinuities. Although this MAC difference does not significantly affect end-to-end inference speed on a high-performance GPU (e.g., RTX 4090), the reduced computational burden of the Haar wavelet provides a clear efficiency advantage for resource-constrained edge hardware without dedicated floating-point units.

#### 4.6.3. Impact of Sampling Strategy

To justify the necessity of the Greedy Coreset algorithm (K-Center) [4], we conducted a comparative analysis against a Naive Random Sampling strategy. Unlike the random approach, which blindly selects features, the Greedy algorithm explicitly identifies the manifold boundaries of the feature space.

Table 5 demonstrates the consistent superiority of our method. While Random Sampling shows instability in structural categories, our Greedy Coreset maintains robust performance across all domains. This advantage is most evident at the 1% memory regime, where the performance gap widens significantly. For instance, in structural categories like *Screw*, Random Sampling degrades to 69.07%, whereas our method maintains a high accuracy of 89.20%, proving its efficiency in preserving critical boundary features.

Selection of Sampling Ratio: Interestingly, our method achieves its peak average accuracy at 1% memory (97.82%). Based on these results, we propose the 1% sampling ratio as the optimal configuration. It offers the best balance between extreme memory efficiency and robust detection performance, effectively discarding redundant features while retaining critical boundary information.

## 5. Discussion

### 5.1. Mechanism of Defect Separation

While AUROC scores indicate the ranking performance, they do not reveal the *confidence margin* of the prediction. To quantify this, we analyzed the Anomaly Score Gap, defined as the difference between the average defect score and the average normal score.

Table 6 presents the aggregated results. Previously, it was hypothesized that semantic context might dilute high-frequency details, potentially harming texture inspection. However, our rigorous analysis reveals that the Semantic Module improves the margin across both domains.

First, the Object Group shows a robust improvement (+8.7%), with the semantic context playing a critical role in structurally complex categories. Notably, *Screw* exhibits a dramatic recovery (+859.7%), where the baseline (Gap ≈ 0.001) failed to distinguish defects, but WEDGE-Net successfully secured a discriminative margin. *Capsule* also shows a significant gain (+22.6%), confirming that semantic guidance reinforces structural awareness.

Contrary to the trade-off concern, the Texture Group also demonstrates a consistent improvement (+4.3%). As seen in *Tile* (+3.9%) and *Wood* (+9.0%), the semantic module aids in suppressing noise in normal patterns rather than obscuring defects. This confirms that WEDGE-Net enhances global discriminability without compromising local sensitivity.

### 5.2. Limitations and Future Works

Despite robust performance in noise-heavy environments, WEDGE-Net exhibits a limitation in retaining high-frequency details, as observed in the *Screw* and *Grid* categories. This is an inherent trade-off of using the Discrete Wavelet Transform (DWT) for compression and denoising. While WEDGE-Net effectively filters noise in aligned objects, its wavelet-driven stream exhibits sensitivity to rotation-variant categories (e.g., *Screw*). To quantitatively assess this, we conducted an experiment applying rotation-based data augmentation. While augmentation yielded a marginal performance improvement (+1.03% AUROC at a 1% coreset), it inherently required a four-fold increase in the memory bank size, which caused the inference speed to drop by nearly half (from 686.5 FPS to 372.85 FPS). Because prioritizing ultra-fast inference is the core objective of our edge-centric design, we concluded that maintaining a highly compressed memory bank without augmentation provides a more practical trade-off for real-world deployment.

While the Semantic Module partially compensates for this loss, fine-grained defects (e.g., subtle thread damage) may still be over-smoothed. Future work could address this by introducing an adaptive DWT mechanism, which dynamically adjusts the frequency filtering level based on the object’s texture complexity, or by integrating a lightweight high-frequency residual stream.

Furthermore, despite the significant efficiency gains from memory compression, we acknowledge that deploying a ResNet-50-based architecture (requiring approximately 3.96G MACs for feature extraction) directly to strictly resource-constrained edge devices, such as microcontrollers, remains computationally challenging. However, in many memory-based anomaly detection frameworks—particularly in practical industrial environments utilizing Industrial PCs (IPCs) or when large memory banks are employed—the k-NN search stage can become a significant computational bottleneck due to extensive memory access and distance computations. By achieving an extreme 1% memory compression, WEDGE-Net substantially reduces the number of stored feature vectors compared to conventional settings, thereby alleviating memory bandwidth overhead and reducing distance computation cost. This design choice provides a practical advantage in real-world deployment scenarios where memory access latency is a critical constraint, and can be viewed as a meaningful step toward more edge-aware anomaly detection systems. Future work will focus on integrating ultra-lightweight backbones to achieve true hardware-level edge optimization.

### 5.3. Implications for Next-Generation Manufacturing

As industrial inspection evolves toward Ultra-High Definition (UHD) sensors, the presence of fine-grained background noise (e.g., surface grain, sensor noise) will inevitably increase. Current RGB-based models often struggle to differentiate between high-resolution detail and actual defects. WEDGE-Net’s capability to selectively filter irrelevant frequency components positions it as a promising solution for these next-generation manufacturing environments, offering a practical path toward high-speed, low-memory, and noise-robust edge AI inspection.

## 6. Conclusions

We presented WEDGE-Net, a memory-efficient anomaly detection framework designed for robust industrial inspection. By leveraging the Discrete Wavelet Transform, our proposed architecture achieved competitive performance on structural and well-aligned textural categories while maintaining a compact memory footprint. This optimized configuration enables a 2.1× increase in inference speed compared to the memory-optimized baseline (and over 10× compared to full-memory models), making it a highly practical and scalable solution for real-time edge deployment in industrial environments.

## Figures and Tables

**Table 1:** Main Results and Memory Efficiency on MVTec AD. We compare WEDGE-Net across varying memory ratios against the optimized PatchCore baseline. The 0.1% ratio serves as a stress test to validate feature robustness. While extreme compression (0.1%) leads to performance drops in complex categories like *Screw*, the 1% setting maintains performance parity with full-memory configurations, particularly in our target domain (e.g., *Transistor*, *Bottle*), confirming that 1% is the optimal efficiency sweet spot.

Category	Type	PatchCore (SOTA)	WEDGE-Net (Ours)			
		*(Official Reported)*	100%	10%	1%	0.1%
*Target Domain (Aligned Structure & Robustness)*						
Tile	Texture	98.7	98.95	98.81	**98.77**	98.38
Cable	Structural	99.5	99.78	99.74	**99.66**	97.40
Transistor	Structural	100.0	100.00	100.00	**100.00**	99.96
Bottle	Structural	100.0	100.00	100.00	**100.00**	100.00
*Texture Classes*						
Carpet	Texture	**98.7**	98.35	98.27	97.95	96.19
Grid	Texture	**98.2**	89.72	90.89	91.14	83.12
Leather	Texture	100.0	100.00	100.00	**100.00**	100.00
Wood	Texture	**99.2**	98.51	98.33	98.51	97.98
Zipper	Texture	99.4	99.05	99.24	**99.45**	99.55
*Structure Classes*						
Capsule	Structural	**98.1**	95.37	95.17	96.33	90.23
Hazelnut	Structural	100.0	100.00	100.00	**100.00**	99.96
Metal Nut	Structural	**100.0**	99.90	99.76	99.76	98.34
Pill	Structural	96.6	96.45	96.29	**96.75**	95.36
Screw	Structural	**98.1**	86.74	88.03	89.20	69.62
Toothbrush	Structural	**100.0**	100.00	100.00	99.72	93.89
**Average**	-	**99.1**	97.52	97.64	97.82	94.67

**Table 2:** Inference Efficiency Comparison on RTX 4090. WEDGE-Net (1%) operates at 686.5 FPS. This efficiency gain is primarily achieved by reducing the memory bank size to 1% without compromising detection accuracy.

Method	Backbone	Params	Memory	Latency (ms)	FPS
PatchCore	Wide ResNet-50	68 M	100%	15.3	65.4
PatchCore	Wide ResNet-50	68 M	10%	3.0	328.9
WEDGE-Net (Ours)	ResNet-50	**25 M**	**1%**	**1.46**	**686.5**

**Table 3:** Impact of the Semantic Module (10% Memory). We analyze the contribution of the Semantic Module by comparing the Frequency-Only Reference (Semantic OFF) against the full WEDGE-Net (Semantic ON). While the reference model effectively filters noise (e.g., *Tile*), it lacks the global context required for complex structures. The Semantic Module recovers this semantic integrity, yielding significant gains in object categories like *Screw*.

Model Variant	Module Status	Noise/Texture		Object/Structure		Key Characteristic
		*Tile*	*Transistor*	*Screw*	*Capsule*	
Frequency-Only	Semantic OFF	**99.42%**	100.00%	71.45%	93.94%	Noise Robustness
WEDGE-Net	Semantic ON	98.81%	100.00%	**88.03%**	**95.17%**	**+ Global Semantics**
Performance Gain		−0.61%	0.00%	**+16.58%**	**+1.23%**	**Semantic Recovery**

**Table 4:** Comparison of Wavelet Kernels. We compare the Haar and Biorthogonal 2.2 wavelets. Haar requires significantly lower computational cost (12.85 million vs. 80.28 million MACs in the DWT stream). Despite its simplicity, Haar maintains robust performance across all memory ratios (e.g., a marginal +0.87% boost at 1% memory) by effectively preserving sharp structural discontinuities. Given that Haar offers a 6.2× reduction in arithmetic complexity without sacrificing accuracy, it is a well-suited choice for industrial edge deployment.

Wavelet Type	DWT Cost (Million MACs)	Global Average AUROC (%)		
		100% Data	10% Data	1% Data
Haar (Default)	12.85	**97.52**	**97.64**	**97.82**
Biorthogonal 2.2	80.28	96.42	96.57	96.95
Difference	−67.43 (6.2× less)	+1.10	+1.07	+0.87

**Table 5:** Impact of Sampling Strategy. We present the complete breakdown of AUROC scores across all 15 MVTec AD categories. The best performance in each row is highlighted in bold. Our Greedy Coreset (K-Center) demonstrates superior stability compared to Random Sampling. Notably, our method achieves its peak performance at 1% memory (97.82%), significantly outperforming Random Sampling (94.23%) and preventing performance degradation in structural categories like *Grid* and *Screw*.

Category	10% Memory		1% Memory	
	Random	Greedy (Ours)	Random	Greedy (Ours)
*Texture Classes*				
Carpet	**98.35**	98.27	97.79	**97.95**
Grid	**91.73**	90.89	83.38	**91.14**
Leather	**100.0**	**100.0**	**100.0**	**100.0**
Tile	**99.28**	98.81	**99.13**	98.77
Wood	98.07	**98.33**	97.46	**98.51**
*Texture Avg.*	**97.49**	97.26	95.55	**97.27**
*Object Classes*				
Bottle	**100.0**	**100.0**	**100.0**	**100.0**
Cable	**99.94**	99.74	**99.87**	99.66
Capsule	90.47	**95.17**	84.32	**96.33**
Hazelnut	**100.0**	**100.0**	**100.0**	**100.0**
Metal Nut	99.51	**99.76**	98.78	**99.76**
Pill	94.46	**96.29**	93.64	**96.75**
Screw	71.37	**88.03**	69.07	**89.20**
Toothbrush	**100.0**	**100.0**	93.61	**99.72**
Transistor	99.92	**100.0**	99.83	**100.0**
Zipper	98.40	**99.24**	96.64	**99.45**
*Object Avg.*	95.41	**97.82**	93.58	**98.09**
**Average (All 15)**	96.10	**97.64**	94.23	**97.82**

**Table 6:** Analysis of Anomaly Score Margins (Average across MVTec AD). We aggregated the Anomaly Score Gap across all 15 categories. The Semantic Module universally improves the separation margin. In the Object group, it provides a critical recovery for difficult classes like *Screw* (+859.7%), which the baseline failed to detect. Crucially, the Texture group also sees a performance boost (+4.3%), proving that our design enhances structural robustness without sacrificing textural sensitivity.

Group/Category	Gap (OFF)	Gap (ON)	Change	Interpretation
Object Average (10 classes)	**0.1144**	**0.1244**	**+8.7%**	**Robust Enhancement**
*e.g., Capsule*	0.0622	0.0763	+22.6%	Structural Gain
*e.g., Screw*	0.0010	0.0096	**+859.7%**	**Critical Recovery**
Texture Average (5 classes)	**0.1285**	**0.1340**	**+4.3%**	**Universal Improvement**
*e.g., Tile*	0.1942	0.2018	+3.9%	Consistent Margin
*e.g., Wood*	0.1258	0.1370	+9.0%	Noise Suppression

*Gap = Avg(Defect Score) − Avg(Normal Score). Higher is better.*

## Data Availability

Publicly available datasets were used in this study. The data are available at https://www.mvtec.com/company/research/datasets/mvtec-ad (accessed on 23 March 2026).

## Supplementary Materials

The following supporting information can be downloaded at: https://www.mdpi.com/article/10.3390/s26072154/s1, Table S1: Pixel-level AUROC and PRO scores on the MVTec AD dataset at 1% memory compression; Table S2: Inference speed (FPS) comparison under a CPU-only environment (Intel Core i9-14900K); Table S3: Trade-off analysis of rotation-based data augmentation on the Screw category at 1% memory compression.
