# Supplementary material for: WEDGE-Net: Wavelet-Driven Memory-Efficient Anomaly Detection for Industrial Edge Computing
Source: Sensors (Basel). 2026 Mar 31;26(7):2154. doi: 10.3390/s26072154 (PMC13074859; doi:10.3390/s26072154)
Supplement: Supplementary file 1 [file sensors-26-02154-s001.zip › sensors-4182732-supplementary.pdf]

Supplementary Material for WEDGE-Net: Wavelet-Driven Memory-Efficient Anomaly Detection for Industrial Edge Computing

Section S1: Pixel-level Evaluation for Anomaly Localization

While the primary objective of WEDGE-Net is image-level binary decision (Go/No-Go inspection), we additionally provide pixel-level evaluation metrics to assess the model's localization capability. Table S1 reports the pixel-level AUROC and Per-Region Overlap (PRO) scores across all 15 categories of the MVTec AD dataset.

Table S1: Pixel-level AUROC and PRO scores on the MVTec AD dataset (evaluated at 1% memory compression). All values are reported in percentage (%).

Category	Pixel AUROC	PRO Score
Bottle	98.05	84.89
Cable	97.88	82.04
Capsule	97.80	72.75
Carpet	98.39	88.57
Grid	93.51	76.32
Hazelnut	98.59	93.41
Leather	98.92	90.77
Metal Nut	95.97	74.55
Pill	98.59	87.92
Screw	93.28	76.22
Tile	94.38	78.06
Toothbrush	97.93	70.54
Transistor	96.81	86.46
Wood	92.54	76.57
Zipper	95.37	76.09
Average	96.53	81.01

Section S2: CPU Inference Benchmarking for Edge Deployment

To evaluate the practical deployment feasibility of WEDGE-Net in Industrial PC (IPC) environments without dedicated GPU acceleration, we conducted inference benchmarking on a CPU-only setup (Intel Core i9-14900K).

In CPU environments, the computational cost of the backbone network often becomes a major factor affecting inference speed. The comparative model utilizes a WideResNet-50 backbone (approximately 11.4G MACs). As a result, even when its memory bank is reduced to 1% to mitigate the cost of k-NN search, its inference speed remains around 33.4 FPS.

In contrast, WEDGE-Net employs a lighter ResNet-50 backbone (approximately 4.0G MACs). As shown in Table S2, this architectural design, combined with memory reduction, helps alleviate computational overhead and enables faster inference speeds (up to 55.5 FPS at 0.1% memory), making it suitable for practical industrial inspection scenarios.

Table S2: Inference speed (FPS) comparison under a CPU-only environment (Intel Core i9-14900K).

Method	Backbone	Memory Ratio	Average FPS
Comparative Model (PatchCore)	Wide ResNet-50	10%	9.8
WEDGE-Net (Ours)	ResNet-50	10%	9.3
Comparative Model (PatchCore)	Wide ResNet-50	1%	33.4
WEDGE-Net (Ours)	ResNet-50	1%	39.4
WEDGE-Net (Ours)	ResNet-50	0.1%	55.5

Section S3: Quantitative Analysis of Rotation-based Data Augmentation

WEDGE-Net exhibits sensitivity to rotation-variant structural objects, which originates from the directional characteristics of the wavelet-driven stream. To quantitatively assess this limitation, we conducted an experiment on the Screw category by applying rotation-based data augmentation $(0°, 90°, 180°, 270°)$.

Table S3 quantitatively demonstrates the trade-off between the marginal improvement in detection accuracy and the substantial degradation in inference speed, which results from the increased memory bank size required to accommodate the augmented samples.

Table S3: Trade-off analysis of rotation-based data augmentation on the Screw category (evaluated at 1% memory compression).

Setting	Image AUROC (%)	Memory Size (Vectors)	Inference Speed (FPS)
Without Augmentation (Default)	89.20	∼2500	686.50
With Rotation Augmentation	90.37	∼10000	372.85
